# PEth as a marker of alcohol consumption in relation to arterial cardiovascular risk factors

**DOI:** 10.1080/02813432.2026.2759986

**Published:** 2026-11-12

**Authors:** Anders Sundström, Magdalena Johansson, Simon Vikström, Jonas Andersson, Marcus M. Lind

**Affiliations:** Department of Public Health and Clinical Medicine, Umeå University, Umeå, Sweden

**Keywords:** PEth, phosphatidyl ethanol, cardiovascular risk factors, alcohol, cross sectional studies

## Abstract

**Background:**

Alcohol has well-known detrimental effects on health. Phosphatidyl ethanol 16:0/18:1 (PEth) is a highly sensitive biomarker of alcohol consumption used in primary healthcare. The relationship between PEth concentrations and cardiovascular risk factors remains unclear. Our hypothesis was that high PEth concentrations would be associated with an unfavourable cardiovascular risk factor profile. This study aimed to investigate the associations between PEth concentrations and arterial cardiovascular risk factors.

**Methods:**

This cross-sectional study was based on data from the Västerbotten Intervention Programme and included 2820 individuals. Health examinations were conducted, including questionnaires collecting data on cardiovascular risk factors. PEth concentrations were analysed in packed erythrocytes from blood samples collected between 1985 and 2014 and stored at −80 °C until analysis. Participants were categorised into four groups. Logistic regression analysis was used to calculate odds ratios (ORs) with 95% confidence intervals (CIs) for the association between PEth concentrations and arterial cardiovascular risk factors.

**Results:**

The median age of participants was 50.2 years, and the median BMI was 25.9. High PEth concentrations (>0.30 μmol/L) were observed in 8.9% of participants and were associated with hypertension (OR 1.58 [95% CI 1.14–2.18]), diabetes (OR 1.89 [95% CI 1.08–3.28]), male sex (OR 2.58 [95% CI 1.90–3.50]), ever smoking (OR 5.31 [95% CI 3.75–7.52]) and lower education level (OR 2.06 [95% CI 1.32–3.22]) compared with PEth <0.01 μmol/L.

**Conclusions:**

High PEth concentrations were associated with established risk factors for arterial cardiovascular disease. These findings should encourage a broader clinical focus when managing patients with elevated PEth concentrations.

## Introduction

Alcohol has well-known harmful effects on health. Globally in 2019, 2.6 million deaths were attributable to alcohol consumption, representing 4.7% of all deaths in that year [1]. High alcohol intake is associated with cancer, injury and increased mortality [2]. Detecting excessive alcohol consumption in primary healthcare remains challenging, particularly as many patients underreport their alcohol use [3]. In recent years, however, certain blood biomarkers also referred to as alcohol markers, have been introduced to aid clinical assessment. Phosphatidyl ethanol 16:0/18:1 (PEth) is an alcohol biomarker currently used in primary healthcare settings due to its high sensitivity [4–7]. PEth can be used by general practitioners to complement a patient’s drinking history and estimate alcohol consumption during the preceding two weeks [8,9]. PEth complements established screening forms. A previous study has shown that using only AUDIT C may result in missing more than 25% of patients with an elevated PEth value >0.3 μmol/L [10].

In primary healthcare, high alcohol consumption is common, and interventions aimed at reducing patients’ alcohol intake can be implemented. Alcohol consumption is well documented to be associated with arterial cardiovascular risk factors such as hypertension [11] and diabetes [12]. However, the relationship between PEth concentrations and arterial cardiovascular risk factors has not been well studied. It is possible that an elevated PEth concentration may provide general practitioners with information beyond a patient’s alcohol consumption alone.

In this cross-sectional study, we aimed to investigate the potential associations between PEth and arterial cardiovascular risk factors.

## Materials and methods

### Study population

The participants in this cross-sectional study consisted of 2820 individuals living in Västerbotten County in northern Sweden who participated in the Västerbotten Intervention Programme (VIP) from 1985 to 2014. The VIP is a health examination programme to which all inhabitants of Västerbotten County aged 40, 50 and 60 years old are invited; a small number of 30-year-olds were also invited [13]. Half of the participants in this study (1410 individuals) later experienced a venous thromboembolic event [14], while the remaining participants were matched controls. Cases and controls were nested within the VIP health examination programme; however, all data used in the present analyses, including blood samples for PEth analysis, were collected before the venous thromboembolic event.

### Cardiovascular risk factors

All the 2820 participants underwent a health examination that included measurements of weight, height and blood pressure. Hypertension was defined as systolic blood pressure ≥140 mmHg and/or diastolic blood pressure ≥90 mmHg, or the use of antihypertensive medication. Body mass index (BMI) was calculated using the formula: weight (kg)/height^2^ (m^2^). Participants with a BMI ≥30 were categorized as obese. Questionnaires regarding smoking status, education level, antihypertensive treatment and exercise habits were completed. Participants were categorized as never smokers or ever smokers (including current, former and occasional smokers). Participants with previously diagnosed diabetes, fasting plasma glucose ≥7.0 mmol/L and/or 2 h plasma glucose ≥12.2 mmol/L were categorized as having diabetes. Education level was categorized as higher education (above secondary school) or not having higher education. Physical activity was categorized as exercising ≥1 time/week or <1 time/week.

### PEth analysis

Venous blood samples were collected after 5 min of rest with minimal stasis following a minimum 4 h of fasting. After collection, samples were centrifuged and separated into plasma, buffy coat and packed erythrocytes, which were kept frozen at −80 °C until analysis. Liquid chromatography–tandem mass spectrometry was used to analyse PEth concentrations in packed erythrocytes. As the samples analysed in this study consisted of packed erythrocytes, the sample volume was adjusted to account for the mean male haematocrit of 45%. Therefore, 45 µL of packed erythrocytes was analysed instead of the standard sample volume of 100 µL of whole blood used for PEth analysis [15]. The method had a lower limit of detection of 0.01 μmol/L, which allows for quantification at lower concentrations than the standard reporting cutoff of 0.05 μmol/L used in Swedish clinical practice. PEth 16:0/18:1 has been found to be stable in erythrocytes when stored at −80 °C, for up to 19 years without significant degradation compared to samples stored for only 1 year [15]. Another study investigating PEth 16:0/18:1 concludes that samples should be stored at −80 °C to maintain long-term stability [16].

### Statistical methods

Baseline characteristics were reported as numbers and percentages for categorical variables, and as medians with first and third quartiles for continuous variables. PEth was treated as a categorical variable. The categories used were PEth <0.01 μmol/L (very low consumption), PEth 0.01 μmol/L to <0.05 μmol/L (low consumption), PEth 0.05 μmol/L to 0.30 μmol/L (moderate consumption) and PEth >0.30 μmol/L (high consumption). PEth <0.01 μmol/L was used as the reference category. Odds ratios (ORs) with 95% confidence intervals (CIs) were calculated using logistic regression analysis. Univariable models were initially used to assess associations between PEth categories and hypertension, diabetes, physical activity, hypercholesterolemia, sex, smoking, BMI and education level, respectively. Age- and sex-adjusted models and multivariable models were then constructed. Variables with a *p* value <0.20 in the univariable analyses were included in the multivariable model. Regression analyses were performed for all participants and separately for men and women. Individuals with missing data were excluded from analyses involving that variable. Statistical analyses were conducted using IBM SPSS version 30.0 (Armonk, NY).

### Ethics

At the VIP health examination, all participants provided informed consent to participate in future research. Research was conducted in accordance with the Declaration of Helsinki. This study was approved by the Regional ethics review board, Umeå, Sweden (approval number 06-162M §157/06) and the Swedish Ethical Review Authority (approval number 2025-06813-02).

## Results

A total of 2820 participants were included in the study, 1556 men and 1264 women. Baseline characteristics for all participants and by PEth category are shown in Table 1.

**Table 1.** Baseline characteristics.

	All (*n* = 2820)	PEth <0.01 µmol/L (*n* = 1193)	PEth 0.01–0.049 µmol/L (*n* = 632)	PEth 0.05–0.30 µmol/L (*n* = 745)	PEth >0.30 µmol/L (*n* = 250)
Age, years (median, 1st–3rd quartile)	50.2 (40.8–60.0)	50.6 (49.7–60.0)	50.1 (40.4–59.9)	50.2 (40.4–60.0)	50.2 (49.6–60.0)
Obesity, *n* (%)	447 (16.1)	227 (19.3)	78 (12.5)	99 (13.4)	43 (17.6)
BMI, kg/m^2^ (median, 1st–3rd quartile)	25.9 (23.6–28.6)	26.0 (23.7–29.0)	25.7 (23.4–28.1)	25.9 (23.5–28.3)	26.2 (24.1–28.7)
Ever smoker, *n* (%)	1533 (55.7)	493 (42.8)	346 (55.7)	494 (67.5)	200 (80.6)
Hypertension, *n* (%)	1110 (40.3)	503 (43.3)	204 (33.0)	277 (38.0)	126 (51.4)
Diabetes, *n* (%)	151 (5.4)	67 (5.7)	32 (5.1)	27 (3.7)	25 (10.2)
Physical activity <1/week, *n* (%)	1806 (71.5)	742 (73.2)	417 (70.1)	478 (69.0)	169 (75.4)
No higher education, *n* (%)	2146 (78.8)	908 (80.4)	453 (72.8)	567 (77.9)	218 (89.3)
Male sex, *n* (%)	1556 (55.2)	537 (45.0)	363 (57.4)	485 (65.1)	171 (68.4)

BMI: body mass index.

Obesity, BMI >30.

The median age for all participants was 50.2 years. The mean BMI was 25.9 (23.6–28.6). A non-detectable PEth concentration (<0.01 μmol/L) was observed in 1193 (42.3%) participants, while a PEth concentration indicating high consumption (>0.30 μmol/L) was observed in 250 (8.9%) participants. Complete baseline data, including data stratified by sex, are shown in Appendix Table A1.

Table 2 presents the results from the univariable and multivariable analyses. The highest PEth category (>0.30 μmol/L) was associated with hypertension (OR 1.58 [95% CI 1.14–2.18]), diabetes (OR 1.89 [95% CI 1.08–3.28]), male sex (OR 2.58 [95% CI 1.90–3.50]), ever smoking (OR 5.31 [95% CI 3.75–7.52]) and no higher education (OR 2.06 [95% CI 1.32–3.22]) compared with the lowest PEth category (<0.01 μmol/L). Univariable analyses stratified by sex are shown in Appendix Table A2. No significant associations were observed between PEth categories and physical activity in any group.

**Table 2.** Associations between phosphatidylethanol (PEth) concentrations and cardiovascular risk factors.

	Participants with risk factor/total number of participants, *n*	Univariable model OR (95% CI)	Model adjusted for age and sex 2 OR (95% CI)	Multivariable model^a^ OR (95% CI)
*Hypertension*				
PEth				
<0.01 μmol/L	503/1193	1 (ref.)	1 (ref.)	1 (ref.)
0.01 to <0.05 μmol/L	204/632	0.65 (0.53–0.79)	0.71 (0.57–0.88)	0.77 (0.61–0.98)
0.05–0.30 μmol/L	277/745	0.81 (0.67–0.97)	0.89 (0.73–1.09)	1.05 (0.84–1.31)
>0.30 μmol/L	126/250	1.39 (1.05–1.83)	1.50 (1.12–2.01)	1.58 (1.14–2.18)
*Diabetes*				
PEth				
<0.01 μmol/L	67/1193	1 (ref.)	1 (ref.)	1 (ref.)
0.01 to <0.05 μmol/L	32/632	0.89 (0.58–1.37)	0.97 (0.63–1.51)	1.12 (0.69–1.80)
0.05–0.30 μmol/L	27/745	0.63 (0.40–1.00)	0.69 (0.43–1.10)	0.78 (0.47–1.29)
>0.30 μmol/L	25/250	1.87 (1.15–3.02)	1.97 (1.21–3.22)	1.89 (1.08–3.28)
*Male sex*				
PEth				
<0.01 μmol/L	537/1193	1 (ref.)	1 (ref.)	1 (ref.)
0.01 to <0.05 μmol/L	363/632	1.65 (1.36–2.00)	1.64 (1.35–1.99)	1.62 (1.32–1.98)
0.05–0.30 μmol/L	485/745	2.28 (1.89–2.75)	2.26 (1.87–2.73)	2.25 (1.84–2.75)
>0.30 μmol/L	171/250	2.64 (1.98–3.53)	2.64 (1.97–3.53)	2.58 (1.90–3.50)
*Ever smoker* PEth				
<0.01 μmol/L	493/1193	1 (ref.)	1 (ref.)	1 (ref.)
0.01 to <0.05 μmol/L	346/632	1.68 (1.38–2.04)	1.69 (1.39–2.07)	1.72 (1.40–2.11)
0.05–0.30 μmol/L	494/745	2.77 (2.28–3.36)	2.79 (2.29–3.40)	2.78 (2.27–3.41)
>0.30 μmol/L	200/250	5.56 (3.97–7.78)	5.56 (3.97–7.80)	5.31 (3.75–7.52)
*Obesity*				
PEth				
<0.01 μmol/L	227/1193	1 (ref.)	1 (ref.)	1 (ref.)
0.01 to <0.05 μmol/L	78/632	0.60 (0.45–0.79)	0.62 (0.47–0.82)	0.70 (0.52–0.94)
0.05–0.30 μmol/L	99/745	0.65 (0.50–0.84)	0.69 (0.53–0.89)	0.72 (0.55–0.95)
>0.30 μmol/L	43/250	0.89 (0.62–1.28)	0.95 (0.66–1.37)	0.71 (0.48–1.06)
*No higher education*				
PEth				
<0.01 μmol/L	908/1193	1 (ref.)	1 (ref.)	1 (ref.)
0.01 to <0.05 μmol/L	453/632	0.66 (0.52–0.83)	0.67 (0.53–0.85)	0.70 (0.55–0.88)
0.05–0.30 μmol/L	567/745	0.86 (0.69–1.08)	0.87 (0.69–1.10)	0.89 (0.70–1.13)
>0.30 μmol/L	218/250	2.05 (1.33–3.16)	2.00 (1.29–3.09)	2.06 (1.32–3.22)

CI: confidence interval; OR: odds ratio.

^a^ Adjusted for hypertension, diabetes, gender, smoking, BMI, education and age. A *p* value <0.05 was considered statistically significant. Analysis was made on packed erythrocytes and adjusted to reflect whole blood levels, as described in ‘Materials and methods’ section and reference [15].

## Discussion

In this study, we found that high PEth concentrations were associated with established risk factors for arterial cardiovascular disease. Hypertension, diabetes, ever smoking, male sex and no higher education were all more common among individuals with high PEth concentrations.

Overall, the highest PEth concentration was associated with a less favourable cardiovascular risk factor profile. However, for hypertension and education, we observed that the group with PEth concentrations of 0.01 to <0.05 μmol/L had a lower risk of hypertension and a higher probability of higher education than participants with the lowest PEth concentrations. These findings, particularly for hypertension are not consistent with previous studies. One systematic review and one meta-analysis have demonstrated an increased risk of hypertension even at low levels of alcohol consumption (<20.0 g/day) [17], and that any alcohol consumption was associated with an increased risk of hypertension in men [18]. It is possible that our findings do not reflect a true effect of alcohol intake but rather residual confounding in the group with low to moderate alcohol consumption. A higher level of education could be associated with modest alcohol consumption but also with an overall beneficial lifestyle, which in turn could have a beneficial effect on blood pressure. This is consistent with previous suggestions from a systematic review and meta-analysis stating that multiple confounding factors may explain the apparent cardiovascular benefits associated with low alcohol consumption [19].

For diabetes, low to moderate alcohol consumption was associated with a lower OR compared with no or high intake, but the association was not statistically significant and may reflect residual confounding. A previous meta-analysis investigating the relationship between alcohol consumption and risk of type 2 diabetes found a U-shaped relationship between the two [12]. Another meta-analysis showed that low to moderate alcohol consumption was associated with a lower risk of type 2 diabetes, which is consistent with our findings; however, it also reported that heavy alcohol consumption had no effect on the incidence of type 2 diabetes [20], which is in contrast to our results.

Somewhat surprisingly, the OR for obesity was significantly lower in the groups with low and moderate PEth concentrations compared to the group with non-detectable PEth concentrations in our material. For participants with high PEth concentrations, we found no significant differences. A systematic review and meta-analysis investigating the relationship between alcohol intake and overweight/obesity reported that heavy alcohol consumption was associated with increased odds of being overweight or obese compared with non-drinkers or light drinkers [21], which contrasts to our findings. Similarly, a cross-sectional study based on Irish health survey data found that harmful alcohol consumption (defined as an AUDIT-C ≥ 5) was significantly associated with overweight and obesity (BMI ≥ 25.0) [22], also in contrast to our results. However, in the present study, obesity was defined using a BMI ≥ 30.0, which limits direct comparability between studies. It is possible that individuals in the highest PEth category in our study may have had lower overall alcohol consumption than high consumers in the studies by Golzarand et al. [21] and AlKalbani and Murrin [22]. This relatively lower level of alcohol intake could explain why no similar association between alcohol consumption and BMI was observed in our study.

A linear association was observed between increasing PEth concentrations and being an ever smoker. This is consistent with previous studies suggesting a significant association between smoking status and alcohol consumption [23].

Strengths of this study include that the study population was derived from a population-based health screening programme (VIP) in which individuals are systematically invited to participate. The health examinations are standardized and conducted according to an established protocol. Previous research has shown only small differences between participants and non-participants in the VIP programme [24], which strengthens the validity of using VIP data. However, individuals with high alcohol consumption may be less likely to attend the health examination, which could introduce selection bias. The sensitive method used to analyse PEth, with a limit of quantification of 0.01 μmol/L, is another strength of this study. Furthermore, the fact that PEth is only formed in the presence of alcohol and has substantially greater specificity for alcohol exposure than many traditional indirect alcohol biomarkers adds to the strengths of this study.

A limitation of this study is its cross-sectional design, which precludes conclusions about causality. The study population consisted of cases and matched controls originally selected for a study of venous thromboembolic events; however, all data were collected prior to any such events. Therefore, this is not considered to have influenced the results of the present study. While the sampling strategy could affect the distribution of cardiovascular risk factors due to the overlap between arterial and VTE risk factors, we do not believe this inherently biases the associations between PEth and the cardiovascular risk factors. To test this formally, we performed a sensitivity analysis stratified by case-referent status, which showed that the findings remained unaffected, with the natural reservation of a minor decrease in statistical power. It is however also not possible to rule out that there is an influence to some extent, as risk factors for arterial cardiovascular disease and risk factors for venous thromboembolism overlap. In our study, we did not adjust for cancer prevalence, which is associated with high alcohol intake and low BMI. This could in theory, to some extent, have influenced our results regarding BMI’s association with different PEth concentrations. Another limitation is that the study population was recruited from the same geographical area in northern Sweden, and alcohol drinking habits may vary across regions. Overall cardiovascular risk profiles may also differ between regions. Therefore, the generalizability of this study to other settings with different conditions may be limited. Since PEth analyses in this study were performed on packed erythrocytes rather than whole blood, which is standard in clinical practice, the values obtained here should not be considered clinically applicable and should not be used as a basis for clinical decisions. Overall, we observed that high PEth concentrations as a marker of high alcohol consumption, were associated with a more unfavourable cardiovascular risk factor profile compared with low concentrations of PEth, indicating low alcohol consumption. The findings in this study are useful in clinical practice and raises awareness among general practitioners that elevated PEth concentrations are associated with a less favourable cardiovascular risk profile. This in turn, encourages a broader clinical focus beyond alcohol consumption alone when managing patients with elevated PEth concentrations.

## Data Availability

Due to restrictions in the ethical approval, data cannot be made publicly available but is available from the corresponding author at reasonable request.

## Appendix

**Table A1.** Baseline characteristics of the participants, overall and stratified by gender and PEth concentrations.

Section A, overall and by gender	All (*n* = 2820)	Men (*n* = 1556)	Women (*n* = 1264)
Age, years (median, 1st–3rd quartile)	50.2 (40.8–60.0)	50.2 (40.6–60.0)	50.3 (49.6–60.0)
Obesity, *n* (%)	447 (16.1)	217 (14.2)	230 (18.4)
BMI, kg/m^2^ (median, 1st–3rd quartile)	25.9 (23.6–28.6)	26.2 (24.2–28.5)	25.5 (23.0–28.6)
Ever smoker, *n* (%)	1533 (55.7)	881 (57.9)	652 (53.0)
Hypertension, *n* (%)	1110 (40.3)	616 (40.7)	494 (39.8)
Diabetes, *n* (%)	151 (5.4)	80 (5.2)	71 (5.7)
Physical activity <1/week, *n* (%)	1806 (71.5)	1010 (71.0)	796 (72.1)
No higher education, *n* (%)	2146 (78.8)	1211 (79.9)	935 (77.3)
PEth <0.01 μmol/L, *n* (%)	1193 (42.3)	537 (34.5)	656 (51.9)
PEth 0.01–<0.05 μmol/L, *n* (%)	632 (22.4)	363 (23.3)	269 (21.3)
PEth 0.05–0.30 μmol/L, *n* (%)	745 (26.4)	485 (31.2)	260 (20.6)
PEth >0.30 μmol/L, *n* (%)	250 (8.9)	171 (11.0)	79 (6.3)

**Table:**

Section B, men stratified by PEth	All men (*n* = 1556)	PEth <0.01 µmol/L (*n* = 537)	PEth 0.01–0.049 µmol/L (*n* = 363)	PEth 0.05–0.30 µmol/L (*n* = 485)	PEth >0.30 µmol/L (*n* = 171)
Age, years (median, 1st–3rd quartile)	50.2 (40.6–60.0)	50.4 (45.3–60.0)	50.2 (40.5–59.9)	50.2 (40.3–60.0)	50.1 (40.7–60.0)
Obesity, *n* (%)	217 (14.2)	74 (14.1)	43 (12.0)	71 (14.7)	29 (17.5)
BMI, kg/m^2^ (median,1st–3rd quartile)	26.2 (24.2–28.5)	25.8 (23.8–28.4)	26.1 (24.2–28.4)	26.4 (24.2–28.7)	26.5 (24.4–28.8)
Ever smoker, *n* (%)	881 (57.9)	232 (44.4)	197 (55.5)	318 (66.9)	134 (79.3)
Hypertension, *n* (%)	616 (40.7)	219 (42.2)	131 (36.8)	182 (38.6)	84 (50.3)
Diabetes, *n* (%)	80 (5.2)	29 (5.5)	18 (5.0)	17 (3.6)	16 (9.5)
Physical activity <1/week, *n* (%)	1010 (71.0)	343 (71.3)	240 (71.0)	317 (70.1)	110 (72.8)
No higher education, *n* (%)	1211 (79.9)	424 (82.3)	260 (73.2)	378 (79.4)	149 (88.2)

**Table:**

Section C, women stratified by PEth	All women (*n* = 1264)	PEth <0.01 µmol/L (*n* = 656)	PEth 0.01–0.049 µmol/L (*n* = 269)	PEth 0.05–0.30 µmol/L (*n* = 260)	PEth >0.30 µmol/L (*n* = 79)
Age, years (median, 1st–3rd quartile)	50.3 (49.6–60.0)	59.3 (49.9–60.0)	50.1 (40.3–60.0)	50.2 (49.4–60.0)	50.3 (49.9–60.0)
Obesity, *n* (%)	230 (18.4)	153 (23.5)	35 (13.2)	28 (10.9)	14 (17.9)
BMI, kg/m^2^ (median, 1st–3rd quartile)	25.5 (23.0–28.6)	26.1 (23.5–29.4)	24.8 (22.4–27.6)	24.4 (22.6–27.2)	25.5 (23.3–28.4)
Ever smoker, *n* (%)	652 (53.0)	261 (41.5)	149 (56.0)	176 (68.5)	66 (83.5)
Hypertension, *n* (%)	494 (39.8)	284 (44,1)	73 (27.8)	95 (37.1)	42 (53.8)
Diabetes, *n* (%)	71 (5.7)	38 (5.9)	14 (5.3)	10 (3.9)	9 (11.5)
Physical activity <1/week, *n* (%)	796 (72.1)	399 (74.9)	177 (68.9)	161 (66.8)	59 (80.8)
No higher education, *n* (%)	935 (77.3)	484 (78.7)	193 (72.3)	189 (75.0)	69 (92.0)

BMI: body mass index.

Obesity, BMI >30. Analysis was made on packed erythrocytes and adjusted to reflect whole blood levels, as described in ‘Materials and methods’ section and reference [15].

**Table A2.** Associations between phosphatidylethanol (PEth) concentrations and cardiovascular risk factors in men and women separately.

Section A, men	Participant with risk factor/total number of participants, *n*	Univariable model OR (95% CI)	Model adjusted for age OR (95% CI)	Multivariable model^a^ OR (95% CI)
*Hypertension*				
PEth				
<0.01 μmol/L	219/537	1 (ref.)	1 (ref.)	1 (ref.)
0.01 to <0.05 μmol/L	131/363	0.80 (0.61–1.05)	0.84 (0.63–1.13)	0.78 (0.57–1.07)
0.05–0.30 μmol/L	182/485	0.86 (0.67–1.11)	0.93 (0.71–1.21)	0.94 (0.70–1.26)
>0.30 μmol/L	84/171	1.39 (0.98–1.97)	1.50 (1.04–2.15)	1.40 (0.93–2.09)
*Diabetes*				
Peth				
<0.01 μmol/L	29/537	1 (ref.)	1 (ref.)	1 (ref.)
0.01 to <0.05 μmol/L	18/363	0.91 (0.49–1.66)	0.97 (0.53–1.78)	0.93 (0.48–1.82)
0.05–0.30 μmol/L	17/485	0.64 (0.35–1.18)	0.68 (0.37–1.27)	0.66 (0.34–1.29)
>0.30 μmol/L	16/171	1.80 (0.95–3.41)	1.92 (1.01–3.66)	1.49 (0.72–3.06)
*Ever smoker* PEth				
<0.01 μmol/L	232/537	1 (ref.)	1 (ref.)	1 (ref.)
0.01 to <0.05 μmol/L	197/363	1.56 (1.19–2.04)	1.59 (1.21–2.09)	1.60 (1.20–2.12)
0.05–0.30 μmol/L	318/485	2.53 (1.96–3.28)	2.62 (2.02–3.39)	2.62 (2.00–3.42)
>0.30 μmol/L	134/171	4.79 (3.18–7.21)	4.90 (3.25–7.39)	4.61 (3.03–7.01)
*Obesity*				
Peth				
<0.01 μmol/L	74/537	1 (ref.)	1 (ref.)	1 (ref.)
0.01 to <0.05 μmol/L	43/363	0.83 (0.56–1.24)	0.83 (0.55–1.24)	0.93 (0.60–1.43)
0.05–0.30 μmol/L	71/485	1.05 (0.74–1.50)	1.05 (0.74–1.49)	1.12 (0.76–1.64)
>0.30 μmol/L	29/171	1.29 (0.81–2.06)	1.29 (0.80–2.06)	1.02 (0.61–1.72)
*No higher education*				
Peth				
<0.01 μmol/L	424/537	1 (ref.)	1 (ref.)	1 (ref.)
0.01 to <0.05 μmol/L	260/363	0.59 (0.42–0.81)	0.60 (0.43–0.83)	0.59 (0.42–0.83)
0.05–0.30 μmol/L	378/485	0.83 (0.60–1.14)	0.86 (0.63–1.19)	0.80 (0.58–1.12)
>0.30 μmol/L	149/171	1.60 (0.95–2.69)	1.63 (0.97–2.74)	1.50 (0.87–2.58)

**Table:**

Section B, women	Participant with risk factor/total number of participants, *n*	Univariable model OR (95% CI)	Model adjusted for age OR (95% CI)	Multivariable model^a^ OR (95% CI)
*Hypertension*				
Peth				
<0.01 μmol/L	284/656	1 (ref.)	1 (ref.)	1 (ref.)
0.01 to <0.05 μmol/L	73/269	0.49 (0.36–0.67)	0.57 (0.41–0.80)	0.73 (0.51–1.04)
0.05–0.30 μmol/L	95/260	0.75 (0.56–1.01)	0.88 (0.64–1.22)	1.17 (0.82–1.68)
>0.30 μmol/L	42/79	1.48 (0.92–2.37)	1.59 (0.96–2.65)	1.77 (1.01–3.10)
*Diabetes*				
Peth				
<0.01 μmol/L	38/656	1 (ref.)	1 (ref.)	1 (ref.)
0.01 to <0.05 μmol/L	14/269	0.89 (0.48–1.68)	0.94 (0.50–1.78)	1.18 (0.60–2.36)
0.05–0.30 μmol/L	10/260	0.66 (0.32–1.33)	0.68 (0.33–1.39)	0.85 (0.39–1.88)
>0.30 μmol/L	9/79	2.09 (0.97–4.50)	2.09 (0.97–4.52)	2.45 (1.01–5.91)
*Ever smoker* PEth				
<0.01 μmol/L	261/656	1 (ref.)	1 (ref.)	1 (ref.)
0.01 to <0.05 μmol/L	149/269	1.80 (1.34–2.40)	1.75 (1.31–2.34)	1.83 (1.35–2.48)
0.05–0.30 μmol/L	176/260	3.06 (2.25–4.17)	3.01 (2.22–4.10)	3.08 (2.23–4.26)
>0.30 μmol/L	66/79	7.18 (3.87–13.25)	7.19 (3.88–13.31)	7.60 (3.90–14.80)
*Obesity*				
PEth				
<0.01 μmol/L	153/656	1 (ref.)	1 (ref.)	1 (ref.)
0.01 to <0.05 μmol/L	35/269	0.49 (0.33–0.73)	0.51 (0.34–0.77)	0.56 (0.37–0.86)
0.05–0.30 μmol/L	28/260	0.40 (0.26–0.61)	0.41 (0.26–0.63)	0.42 (0.26–0.66)
>0.30 μmol/L	14/79	0.71 (0.39–1.30)	0.71 (0.39–1.30)	0.40 (0.20–0.81)
*No higher education*				
PEth				
<0.01 μmol/L	484/656	1 (ref.)	1 (ref.)	1 (ref.)
0.01 to <0.05 μmol/L	193/269	0.71 (0.51–0.98)	0.75 (0.54–1.04)	0.74 (0.52–1.05)
0.05–0.30 μmol/L	189/260	0.81 (0.58–1.15)	0.85 (0.60–1.20)	0.80 (0.55–1.15)
>0.30 μmol/L	69/79	3.11 (1.32–7.33)	3.10 (1.31–7.30)	2.70 (1.12–9.65)

CI: confidence interval; OR: odds ratio.

A *p* value <0.05 was considered statistically significant. Analysis was made on packed erythrocytes and adjusted to reflect whole blood levels, as described in ‘Materials and methods’ section and reference [15].

^a^ Adjusted for hypertension, diabetes, gender, smoking, BMI, education and age.
